# The role of exercise and hypoxia on glucose transport and regulation

**DOI:** 10.1007/s00421-023-05135-1

**Published:** 2023-01-23

**Authors:** J. Soo, A. Raman, N. G. Lawler, P. S. R. Goods, L. Deldicque, O. Girard, T. J. Fairchild

**Affiliations:** 1 Australian National Phenome Centre, Health Futures Institute, Murdoch University, Perth, Australia; 2 Discipline of Exercise Science, Murdoch University, Perth, WA 6018 Australia; 3 The Centre for Healthy Aging, Health Futures Institute, Murdoch University, Perth, Australia; 4 Institute of Neuroscience, Université Catholique de Louvain, Louvain-la-Neuve, Belgium; 5 School of Human Sciences (Exercise and Sport Science), The University of Western Australia, Crawley, WA Australia; 6 The Centre for Molecular Medicine and Innovative Therapeutics, Health Futures Institute, Murdoch University, Perth, Australia

**Keywords:** Glucose, Insulin resistance, Hypoxia, Glucose metabolism, Exercise

## Abstract

Muscle glucose transport activity increases with an acute bout of exercise, a process that is accomplished by the translocation of glucose transporters to the plasma membrane. This process remains intact in the skeletal muscle of individuals with insulin resistance and type 2 diabetes mellitus (T2DM). Exercise training is, therefore, an important cornerstone in the management of individuals with T2DM. However, the acute systemic glucose responses to carbohydrate ingestion are often augmented during the early recovery period from exercise, despite increased glucose uptake into skeletal muscle. Accordingly, the first aim of this review is to summarize the knowledge associated with insulin action and glucose uptake in skeletal muscle and apply these to explain the disparate responses between systemic and localized glucose responses post-exercise. Herein, the importance of muscle glycogen depletion and the key glucoregulatory hormones will be discussed. Glucose uptake can also be stimulated independently by hypoxia; therefore, hypoxic training presents as an emerging method for enhancing the effects of exercise on glucose regulation. Thus, the second aim of this review is to discuss the potential for systemic hypoxia to enhance the effects of exercise on glucose regulation.

## Introduction

Maintaining blood glucose concentration within a narrow range is not trivial, with postprandial glycaemic excursions overlaying the fasting blood glucose concentrations. This is particularly evident in type 2 diabetes mellitus (T2DM), where insulin resistance and altered glucose metabolism are a natural consequence of the clinical history. In T2DM, poor glycaemic control is associated with increased risks of cardiovascular disease (Bassuk and Manson 2005; Gayda et al. 2008), renal disease (Ismail et al. 1999), non-traumatic amputations (Reiber et al. 1995) and all-cause mortality (Laukkanen et al. 2013).

Glycaemic control is improved with chronic exercise training (Umpierre et al. 2013; Boulé et al. 2003; Burgomaster et al. 2007; Mikus et al. 2012; Richards et al. 2010; Whyte et al. 2010). Improved glycaemic control in response to exercise training is primarily attributed to improved insulin-mediated glucose disposal (Babraj et al. 2009; Richards et al. 2010; Whyte et al. 2010) rather than lower fasting glucose concentration (MacLeod et al. 2013). Given that the skeletal muscle is the predominant site for insulin-mediated glucose disposal, regular exercise training is thus an important factor for glycaemic control. Importantly, these effects of exercise on glucose transport remain intact in individuals with T2DM. However, whilst the benefits of chronic exercise training on glycaemic control (commonly assessed via glycated haemoglobin [HbA1c]) are well established (Tsukui et al. 2000; Thomas et al. 2006; Umpierre et al. 2011), there is large variability in the magnitude of the response (Boulé et al. 2005). Potential factors contributing to this variability include individual differences (e.g. duration of T2DM; dietary intake), and differences in exercise-related factors such as the volume, type and intensity of exercise. Increases in insulin-stimulated skeletal muscle glucose uptake with exercise is associated with the magnitude of muscle glycogen depletion during exercise (Ivy et al. 1985; Bogardus et al. 1983). Indeed, both insulin- and contraction-mediated glucose transport are influenced by skeletal muscle glycogen concentration (Host et al*.* 1998; Derave et al. 2000; Derave et al. 1999; Kawanaka et al. 1999; Kawanaka et al. 2000). However, glycogen utilization is dependent on exercise being performed at a sufficiently high intensity and/or duration, which is challenging in clinical populations. As such, alternative exercise interventions are continually sought.

Exercising in hypoxia may represent an effective strategy to enhance muscle glycogen utilization and glucose uptake. The relative intensity of exercise is substantially higher when performed in hypoxia than in normal ambient conditions (i.e., normoxia). As such, increases in exercise intensity, which is important for improving glucose regulation, can be undertaken without a substantial increase in workload. Furthermore, hypoxia may stimulate the activation of signalling molecules responsible for glucose transport activity, similar to high-intensity exercise. Exercising in hypoxia may thus promote a synergistic effect on glucose regulation that is safe and effective for individuals with T2DM.

The purpose of this review is to first summarize the existing knowledge related to insulin action garnered from classic in vitro and in situ glucose uptake studies. The effects of exercise and insulin on cellular glucose uptake and the physiological consequences on systemic glucose concentration are then reviewed. Finally, this review aims to explain factors that may influence the mismatch between cellular glucose uptake and systemic glucose concentration following exercise. In this context, systemic changes in plasma glucose post-exercise in relation to the effects of key glucoregulatory hormones will be discussed. Finally, the review will discuss the potential for hypoxia to enhance the effects of exercise on systemic glucose regulation.

## Glucose transport

Cellular uptake of glucose occurs through facilitated diffusion using a carrier protein from the glucose transporter (GLUT) family. There are 14 facilitative glucose transporter proteins encoded in the human genome (Mueckler and Thorens 2013). Of these, glucose transporter type 1 (GLUT-1) is ubiquitously distributed and does not change in response to hormonal or other stimuli (Douen et al. 1990; Holloszy and Hansen 1996). For this reason, GLUT-1 is primarily responsible for glucose transport under basal conditions.

Glucose transporter type 4 (GLUT-4), is present primarily in adipose cells (Garvey et al. 1991; Hussey et al. 2011) and striated (skeletal and cardiac) muscle cells (Olson and Pessin 1996). Of the several glucose transporters including GLUT-1, GLUT-5 and GLUT-12, GLUT-4 is the predominant protein expressed in the skeletal muscle (Stuart et al. 2006). That said, the GLUT-4 protein content varies between muscle fibre types (Henriksen et al. 1990), but can be increased with exercise training, even in individuals with T2DM (O'Gorman et al. 2006). In this instance, GLUT-4 displays membrane trafficking capability (Huang and Czech 2007) that is highly responsive to insulin, muscle contraction and other stimuli (e.g. hypoxia) (Richter et al. 2001). Accordingly, GLUT-4 is likely responsible for the bulk of glucose uptake into the muscle and is, therefore, an important determinant of glucose homeostasis.

**Fig. 1:**
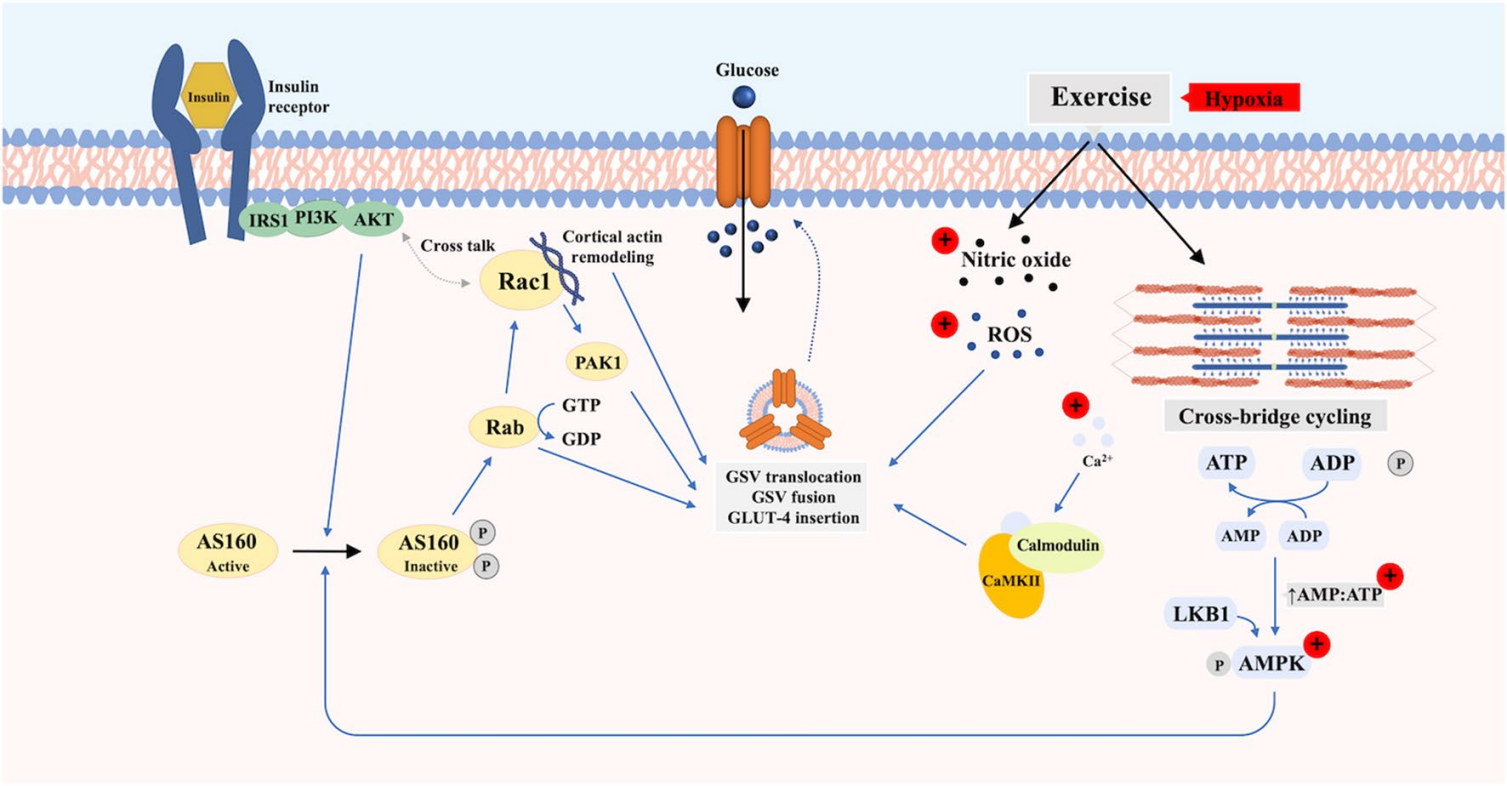
Proposed pathways mediating translocation of GLUT-4 to the cell membrane of skeletal myocyte in response to insulin and exercise/contraction with and without additional hypoxic stimulus. Upon insulin binding, the activated insulin receptor initiates downstream metabolic signalling that recruits diverse substrates, which ultimately leads to the translocation and fusion of the glucose transporter storage vesicle to the cell membrane and insertion of GLUT-4. Exercise and muscle contraction stimulate GLUT-4 translocation and glucose uptake through a distinct mechanism independent of insulin. This mechanism involves changes in cellular energy status, intracellular Ca^2+^ concentration, reactive oxygen species (ROS) and nitric oxide (NO). Hypoxia activates GLUT-4 translocation via similar or overlapping pathways to that of contraction-mediated glucose uptake. The (+) indicates the proposed potentiation of the hypoxia stimulus

Insulin plays a central role in glucose homeostasis through its direct effect on insulin-sensitive tissues, namely, the liver, adipose tissues and skeletal muscle (Petersen and Shulman 2018). The skeletal muscle comprising ~ 40% of total body mass, accounts for ~ 85% of insulin-stimulated glucose disposal (DeFronzo et al. 1981). Insulin binding (Wardzala and Jeanrenaud 1981) and/or muscle contraction (Hirshman et al. 1990; Goodyear et al. 1990a) initiates the translocation of GLUT-4 from its intracellular compartments to the sarcolemma and t-tubules. In this instance, an increased GLUT-4 protein content at the cell membrane ultimately increases the rate of glucose transport (Constable et al. 1988; Goodyear et al*.* 1990b). Specifically, the increase in GLUT-4 content and glucose transport parallels the increased contraction rates (Lund et al. 1995). Combining maximal insulin stimulation (1 mU/ml) and muscle contractions (10 Hz for 5 min) has also been shown to result in a 9.3-fold increase in the GLUT-4 plasma membrane expression in rat *soleus* muscle (Lund et al. 1995). This additive effect in skeletal muscle may be due to the existence of discrete intracellular GLUT-4 pools, which are mobilized via distinct molecular mechanisms (Coderre et al. 1995; Hansen et al. 1998; Richter et al. 1987) (Fig. 1). That said, the molecular mechanisms stimulating GLUT-4 translocation during insulin stimulation and contraction do appear to partially converge at the Rab GTPase-activating protein (GAP) AKT substrate of 160 kDa (AS160 or also known as TBC1D4) and GAP TBC1D1 (Mackenzie and Watt 2016; Sylow et al. 2021). Phosphorylation of TBC1D4/TBC1D1 inactivates Rab-GAP activity regulating vesicle trafficking which in turn, increase the concentration of additional signalling molecules (e.g. Rac1) (Sylow et al. 2013).

Increased rates of glucose transport have been shown to persist into recovery following exhaustive exercise, with the rate of glucose transport reported to be ~ 5.6-, ~ 3.3-, ~ 2.7-, and ~ 1.5-fold higher than pre-exercise (Wallberghenriksson et al. 1988) at 10, 30, 60 and 180 min post-exercise. These findings in rat *epitrochlearis* muscle provided early evidence that glucose uptake after exercise occurs in two phases. An additive effect of insulin and exercise on glucose uptake during the early stages of recovery; followed by an increase in insulin sensitivity and responsiveness during the latter stages of recovery when contraction-mediated glucose uptake had largely been reversed (Wallberghenriksson et al. 1988).

## Insulin sensitivity and responsiveness

Insulin sensitivity refers to the concentration of insulin required to achieve half of its maximal effect on glucose transport (Holloszy 2005). Conversely, insulin responsiveness refers to the rate of glucose transport associated with a maximally effective insulin. In this instance, the measurement of insulin sensitivity post-exercise requires careful interpretation to distinguish between increased insulin action (i.e., latter stage of recovery) and exercise-induced increase in glucose transport that is additive to insulin (early stage of recovery) (Table 1).

**Table 1:** The acute and chronic hormonal responses during exercise with and without hypoxia

Hormones	Protocol	Observations
Insulin	Exercise in NOR	Acute response Insulin concentration decreases (Gyntelberg et al. 1977) during acute prolonged cycling (60% $\dot{V}{\text{O}}_{2\max }$), relative to baseline Insulin concentration increases significantly (and rapidly) during post-exercise recovery (Kjaer et al. 1990; Marliss et al. 1991) Training response Decrease in insulin during exercise is attenuated following exercise training compared to prior training at the same absolute intensity (Gyntelberg et al. 1977; Mendenhall et al. 1994)
	Exercise in NOR vs. Exercise in HYP	Acute response Insulin response to glucose at rest is similar between hypoxia and normoxia (Kelly et al. 2010) Similar insulin response to exercise in hypoxia (~ 2000–4300 m) relative to normoxia (Bouissou et al. 1986; Bailey et al. 2015; Engfred et al. 1994; Matu et al. 2017), whereas insulin response during post-exercise recovery is elevated in severe hypoxia (4300 m) compared to moderate hypoxia (2100 m) and normoxia (Matu et al. 2017) Training response Similar decrease in insulin response during exercise in normoxia following 5 weeks of training in normoxia or hypoxia (Engfred et al. 1994)
Glucagon	Exercise in NOR	Acute response ~ Threefold increase from baseline during prolonged exhaustive treadmill running (i.e., volitional exhaustion) (Galbo et al. 1975) Increase in catecholamine is associated with the rise in glucagon concentration but does not fully account for the glucagon response to prolonged exercise (Galbo et al. 1975, 1976) Training response Increase in glucagon during exercise is blunted following a 10-week (4 d/wk) exercise training (Gyntelberg et al. 1977) The attenuated increase in glucagon is observed during exercise performed at the same relative (Gyntelberg et al. 1977) and absolute intensity (Gyntelberg et al. 1977; Mendenhall et al. 1994; Winder et al. 1979) as pre-training
	Exercise in NOR vs. Exercise in HYP	Acute response No significant differences in glucagon response during a maximal graded exercise that was performed in normoxia and hypoxia at similar relative intensity (Bouissou et al. 1986) Training response Glucagon response to exercise (in normoxia) following 5 weeks of training in normoxia or hypoxia was similar (Engfred et al. 1994)
Epinephrine	Exercise in NOR	Acute responses Plasma epinephrine concentration increases with exercise intensity (Kjaer et al. 1990; Galbo et al. 1975) ~ Threefold and ~ 14 fold increase from baseline during acute exhaustive cycling at 75% (Hartley et al. 1972b) and 100% $\dot{V}{\text{O}}_{2\max }$ (Marliss et al. 1991), respectively Exaggerated epinephrine response to maximal exercise in T2DM compared to healthy individuals (Kjaer et al. 1990) Training responses Attenuated increase in epinephrine response to exercise at the same absolute intensity (as pre-training) following short-term training (10 days, 6d/wk) with minimal changes after further training (12 weeks) (Mendenhall et al. 1994)
	Exercise in NOR vs. Exercise in HYP	Acute responses Resting plasma epinephrine AUC after glucose ingestion is ~ twofold greater in hypoxia compared to normoxia (Kelly et al. 2010) Similar increase in epinephrine response during a maximal graded exercise that was performed in normoxia and hypoxia at similar relative intensity (Bouissou et al. 1986) Training response 5-week exercise training in hypoxia at the same relative or absolute intensity to normoxia did not alter epinephrine response (albeit blunted increase) to exhaustive exercise performed at the same absolute workload before training (Engfred et al. 1994)
Norepinephrine	Exercise in NOR	Acute response ~ Sevenfold and ~ ninefold increase in norepinephrine following continuous (15 min) and intermittent running (3 × 300 m), respectively, (Näveri et al. 1985) compared to baseline ~ 18-fold increase from baseline during exhaustive high-intensity (100% $\dot{V}{\text{O}}_{2\max }$) cycling (Marliss et al. 1991) Exaggerated norepinephrine response to maximal exercise in T2DM compared to healthy individuals (Kjaer et al. 1990) Training response Attenuated increase in epinephrine response to exercise at the same absolute intensity (as pre-training) following short-term training (10 days, 6d/wk), with minimal changes after further training (12 weeks) (Mendenhall et al. 1994)
	Exercise in NOR vs. Exercise in HYP	Acute response Similar norepinephrine response during submaximal exercise in hypoxia relative to normoxia (performed at same relative intensity), but lower response at maximal aerobic capacity (100% $\dot{V}{\text{O}}_{2\max }$) (Bouissou et al. 1986) Training response 5-week exercise training in hypoxia at the same relative and absolute intensity to normoxia did not alter epinephrine response (albeit blunted increase) to exhaustive exercise that was performed at the same absolute workload before training (Engfred et al. 1994)
Growth hormone	Exercise in NOR	Acute response Growth hormone concentration increased in response to exercise (Kjaer et al. 1990; Kindermann et al. 1982) ~ 14-fold increase following prolonged exercise (50 min at the anaerobic threshold of 4 mmol/L) (Kindermann et al. 1982) Training response Growth hormone response to an acute bout of exercise is not altered following 10-week training (at similar relative intensity) of different modalities (running, resistance training, and combined) (Craig et al. 1991)
	Exercise in NOR vs. Exercise in HYP	Acute response Hypoxia exercise (60 min at 60% of *V*O_2max_) induces a similar increase in growth hormone compared to exercise in normoxia at the same relative intensity (Schmidt et al. 1995) Elevated growth hormone concentration immediately after exercise following repeated sprint exercise (30 s efforts) in hypoxia compared to normoxia (Kon et al. 2015) Training response 5-week exercise training in hypoxia at the same relative and absolute intensity as normoxia did not alter growth hormone response to exhaustive exercise that was performed at the same absolute workload before training (Engfred et al. 1994)
Cortisol	Exercise in NOR	Acute response ~ 1.3- and ~ 1.5-fold increase in plasma cortisol concentration following aerobic (50 min of treadmill running at 4 mmol/L) and anaerobic (all-out run to exhaustion at 20 km/h) exercise, respectively (Kindermann et al. 1982) Greater cortisol response with increasing exercising intensity (> ~ 60% $\dot{V}{\text{O}}_{2\max }$), whereas lower exercise intensity elicits minimal changes compared to baseline (Hill et al. 2008) Cortisol response to exercise, when expressed as absolute workload, is lower in trained individuals compared to untrained and moderately trained individuals (Luger et al. 1987) Training response Cortisol response to exhaustive exercise (at the same absolute intensity as pre-training) remains largely unchanged following 7 weeks of exercise training (Hartley et al. 1972b)
	Exercise in NOR vs. Exercise in HYP	Acute response Elevated acute cortisol response in hypoxia compared to normoxia during exercise performed at similar absolute intensity (Sutton 1977), whereas similar response during exercise performed at the same relative intensity (Bouissou et al. 1986) Training response Increase in cortisol in response to exhaustive exercise at 85% $\dot{V}{\text{O}}_{2\max }$ following 5 weeks of exercise training in hypoxia at the same relative and absolute intensity to normoxia (Engfred et al. 1994)
Incretins (postprandial responses)		
GLP-1	Exercise in NOR	Acute response Prior exercise (60 min of treadmill walking at 55–60% $\dot{V}{\text{O}}_{2peak}$) did not alter GLP-1 response to a mixed meal (Heden et al. 2013) or oral fat tolerance test (Dekker et al. 2010) measured 12 and 16 h post-exercise Training response Short-term exercise training (7 consecutive days) decreased fasting plasma GLP-1 concentration and increased acute GLP-1 response to glucose in obese individuals with non-alcoholic fatty liver disease (Kullman et al. 2016)
	Exercise in NOR vs. Exercise in HYP	Acute response Resting and postprandial GLP-1 response were not altered by hypoxia exposure (FiO_2_ ~ 0.15) (Morishima and Goto 2016) GLP-1 response following continuous moderate or high-intensity interval exercise were identical between hypoxia and normoxia (Bailey et al. 2015) Postprandial GLP-1 response was not altered following exercise in hypoxia (compared to normoxia) (Bailey et al. 2015) Training response Postprandial GLP-1 response following exercise training was elevated following 4-week training (3d/wk) in normoxia, but unchanged in hypoxia (Morishima et al. 2014)
GIP	Exercise in NOR	Acute response GIP response remains relatively unchanged during 120 min of running at 60% $\dot{V}{\text{O}}_{2\max }$ (O’Connor et al. 2006), and 3-h cycling at 40% $\dot{V}{\text{O}}_{2\max }$ (Hilsted et al. 1980) Single bout of running 120 min at 60% $\dot{V}{\text{O}}_{2\max }$ (O’Connor et al. 2006) or exercise at 75% $\dot{V}{\text{O}}_{2\max }$ (Blom et al. 1985) until exhaustion attenuates increase in postprandial plasma GIP concentration during the immediate postexercise recovery phase Training response Effect of exercise training on postprandial GIP response remains unclear, partly due to factors including dietary restriction (Kelly et al. 2009; Kahle et al. 1986) and weight loss (Kelly et al. 2009; Solomon et al. 2010)
	Exercise in NOR vs. Exercise in HYP	NA

*AUC* area under curve, *Ex* exercise, *GLP-1* glucagon-like peptide-1, *GIP* gastric inhibitory polypeptide, *HYP* hypoxia, *NA* not applicable, *NOR* normoxia, *T2DM* type 2 diabetes mellitus

The duration of increased insulin sensitivity following exercise may persist from 3 to 48 h and is dependent on the dietary status (Cartee et al. 1989; Gulve et al. 1990; Richter et al. 1982). In rats fed a carbohydrate-free diet post-exercise, the exercise-stimulated increases in glucose uptake (7.8-fold above non-exercised levels) were still largely present 18 h later (3.5-fold increase above non-exercised levels). However, in rats fed a 60% carbohydrate diet, the exercise-stimulated glucose uptake was no longer present at the same time-point (1.1-fold increase above non-exercise levels) (Young et al. 1983). These findings support the observations that insulin-mediated glucose uptake and GLUT4 translocation in the skeletal muscle of rats, are directly associated with muscle glycogen concentration (Host et al. 1998; Derave et al. 2000; Derave et al. 1999; Kawanaka et al. 1999; Kawanaka et al. 2000).

## Glucose transport measurement in humans

The in vivo measurement of glucose transport in humans is challenging and relies on tracer-labelled glucose (such as [^13^C]glucose, [^2^H]glucose) (Zinker et al. 1993a, b). The tracer-labelled glucose is coupled with either tissue biopsies (Roussel et al. 1998), measures of arterio-venous glucose difference (Wahren et al. 1971), or imaging techniques such as dynamic positron emission tomography (Bertoldo et al. 2005) and magnetic resonance spectroscopy (Roden and Shulman 1999) which allow assessment of tissue glucose uptake rates. Using arterio-venous glucose differences, skeletal muscle glucose transport was shown to increase with intensity as well as duration during knee extensions in humans (Kjaer et al. 1990; Holloszy and Narahara 1965; Helge et al. 2003). For instance, glucose uptake rates of 0.05, 0.3, 0.75 and 1.07 mmol/min/thigh were reported at rest, 25%, 65% and 85% of maximal work capacity (Helge et al. 2003). The greater recruitment of total muscle fibres, and in particular fast-twitch fibres, likely underpins the higher rates of glucose uptake at higher work capacities (Katz et al. 1986; Ploug et al. 1984).

## The role of exercise intensity on glucose regulation

Increasing exercise intensity results in greater recruitment of muscle fibres as well as an increased reliance on plasma glucose and muscle glycogen (Coggan 1991; Jeukendrup 2011; Sahlin 1992; Vollestad and Blom 1985) for energy. Furthermore, increased utilization and reduction of muscle glycogen are associated with improved glucose uptake in the skeletal muscle of rats (Host et al. 1998; Derave et al. 2000; Kawanaka et al. 2000) and glucose tolerance in humans (Kang et al. 1996). Hence, it is tenable to expect that high-intensity exercise enhances glucose tolerance. However, when matched for total work, low-moderate intensity exercise (40–50% $\dot{V}{\text{O}}_{{2{\text{peak}}}}$) resulted in a similar GLUT-4 recruitment to high intensity (80% $\dot{V}{\text{O}}_{{2{\text{peak}}}}$) exercise in healthy untrained individuals (Kraniou et al. 2006). Similarly, there were no differences in plasma glucose response to an oral glucose tolerance test (OGTT) performed 1 h and 24 h after a moderate- or high-intensity exercise bout in healthy, middle-aged individuals (Bonen et al. 1998). In contrast, in prediabetic adults the plasma glucose and insulin response to an OGTT were improved 1 h post an isocaloric moderate- or high-intensity exercise bout, although the high-intensity exercise improved insulin sensitivity to a greater degree than the moderate-intensity exercise bout (Rynders et al. 2014).

High-intensity interval exercise/training (HIIE/HIIT) protocols are defined as brief periods of near-maximal efforts (≤ 4 min) that elicit ≥ 80% of maximal heart rate or $\dot{V}{\text{O}}_{2\max }$ interspersed with a short period of rest or lower-intensity exercise (Callahan et al. 2021; MacInnis and Gibala 2017). HIIE results in significant muscle glycogen depletion (Hermansen and Vaage 1977), which would be expected to elicit increased skeletal muscle glucose uptake. Post-meal glucose AUC and ‘time in hyperglycaemia’ during the 24 h following HIIE were reduced in overweight/obese individuals with T2DM (Gillen et al. 2012; Little et al. 2011). In contrast, a continuous moderate-intensity (45 min at 75% $\dot{V}{\text{O}}_{{2{\text{peak}}}}$) cycling has been shown to elicit greater improvements in insulin sensitivity 24 h post-exercise when compared to an interval-based exercise (five 30-s cycling bout performed at 125% $\dot{V}{\text{O}}_{{2{\text{peak}}}}$ interspersed with 4–5 min of rest or 50 W cycling) in overweight/obese individuals (Brestoff et al. 2009). This was due largely to reduced fasting plasma insulin concentration (rather than changes in glucose concentration) and insulin AUC during the OGTT (Brestoff et al. 2009), indicating an improvement in muscle insulin action.

## Changes in systemic plasma glucose post-exercise: a balance between the rate of glucose appearance and disappearance

While acute exercise may increase the uptake of glucose, it should be noted that systemic plasma glucose concentration ultimately reflects the balance between the rate of glucose appearance (*R*_a_) or entry, and the rate of glucose disappearance (*R*_d_) or exit from the circulation. Glucose *R*_a_ in the fasted state is principally governed by total hepatic glucose release. Glucose *R*_a_ in the postprandial period is governed by total hepatic glucose release and the glucose remaining in the portal vein (dietary glucose absorbed from the small intestine) following hepatic first-pass. The *R*_d_ is the sum of glucose uptake by all cells of the body. In this instance, the balance between *R*_a_ and *R*_*d*_ becomes more complex during exercise since both *R*_a_ and *R*_d_ are affected. Specifically, acute exercise may (i) increase—in orders of magnitude—glucose uptake into the exercised/contracted muscles (Sylow et al. 2017); (ii) decrease in insulin response and glucose uptake in tissues other than the contracting musculature (i.e., reduced *R*_d_ in inactive tissues); (iii) increase net hepatic glucose release which remains elevated for approximately 30 min after exercise; and (iv) decrease the rate of glucose absorption from the small intestine (Knudsen et al. 2014). Furthermore, during high-intensity exercise, a host of glucoregulatory hormones (described below), rather than insulin per se, become the key regulators of glucose regulation.

## Glucoregulatory effects of key hormones associated with exercise: epinephrine, glucagon, growth hormone and cortisol

**Fig. 2:**
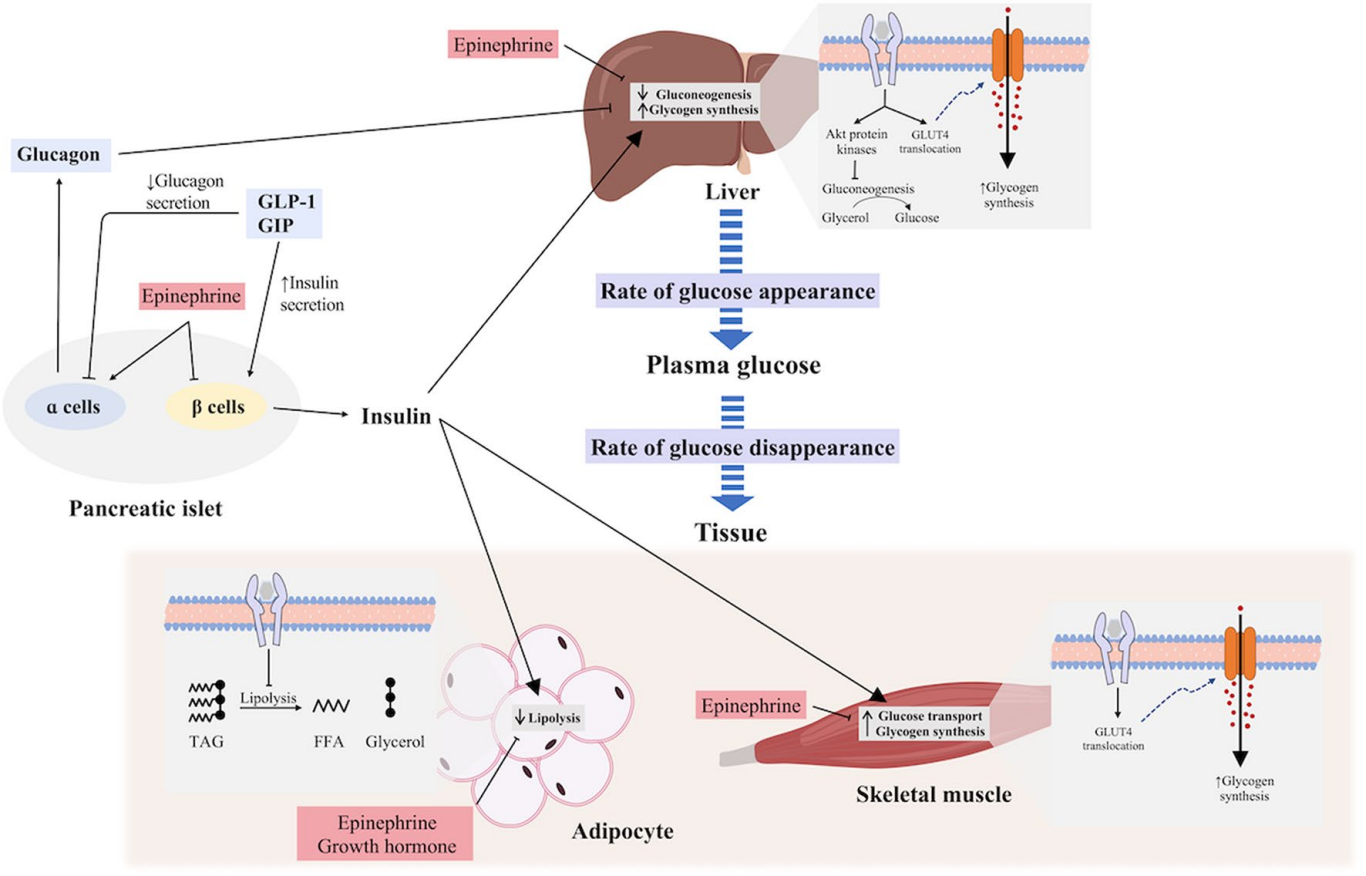
Glucoregulatory effects of key hormones controlling plasma glucose concentration. Effect of insulin on the liver, adipose tissue and skeletal muscle pathways are demonstrated, along with counter-regulatory effects of epinephrine and norepinephrine, growth hormone and glucagon

Increased hepatic glucose output is essential to sustaining prolonged exercise capacity and preventing hypoglycaemia (Trefts et al. 2015; Wasserman 2009), with the increased glucose output continuing for up to 30–60 min into the recovery period (Knudsen et al. 2014). This increased hepatic glucose output coincides with increases in glucagon, catecholamine and cortisol concentrations and decreases in insulin concentration (Wasserman 2009; Kindermann et al. 1982). Of these hormones, single infusions of epinephrine and glucagon during resting conditions are associated with increases in plasma glucose, with glucagon increasing hepatic glucose output to the greatest level, while cortisol appears to have negligible effect in isolation (Eigler et al. 1979; Kjaer et al. 1990; Kjaer 1998) (Fig. 2). Glucagon, in concert with the associated decrease in insulin concentration, likely plays the greatest role in increasing hepatic glucose release during exercise (Hirsch et al. 1991; Wasserman et al. 1989). In contrast, the influences of catecholamines, and specifically epinephrine on increased hepatic glucose output, appear minimal (Wasserman et al. 1990; Coker et al. 2000; Moates et al. 1988). Several hormones appear to act synergistically on hepatic glucose production, with the addition of epinephrine and glucagon being greater than either alone, and the addition of cortisol prolonging the action of these hormones on the liver (Eigler et al. 1979).

While the effects of glucagon may be largely limited to the liver, epinephrine can play important roles in *R*_d_ (Sherwin et al. 1980). In perfused rat muscles, epinephrine (25 nM) significantly increased GLUT-4 content and glucose transport (22–48%), with the greatest increase occurring in oxidative muscle (red *gastrocnemius* and *soleus*) (Han and Bonen 1998). However, infusion of epinephrine (25 nM) has been shown to partially inhibit the insulin-stimulated glucose transport when compared to insulin infusion (20 mU/ml) alone, despite the persistent increase in GLUT-4 plasma membrane content (Han and Bonen 1998). Subsequent work found that the inhibition of glucose transport by epinephrine (24 nM) persists at moderate (50 mU/ml), but not at a high physiological concentration of insulin (100 mU/ml), even when the concentration of epinephrine was increased to 500 nM (Hunt and Ivy 2002). The underlying mechanisms associated with the downregulation of insulin-stimulated glucose transport with epinephrine is likely associated with a decreased activity of insulin receptor substrate-1 associated phosphatidylinositol 3-kinase (PI3K) (Hunt and Ivy 2002).

Circulating levels of growth hormone (GH) are increased with exercise (Hirsch et al. 1991), with the magnitude of change being dependent on exercise duration and intensity (Hartley et al. 1972a; Kindermann et al. 1982). The role of GH in glucose regulation is complex and appears to occur in two phases with an initial, transient, insulin-like response followed by an anti-insulin response in the hours thereafter (Rizza et al. 1982; Smith et al. 1997; Svensson et al. 2002). The anti-insulin effects of GH include the promotion of gluconeogenesis and hepatic glucose release (Schwarz et al. 2002; Svensson et al. 2002), although these effects seem minimal (Kjaer 1998). GH was shown to reduce glucose uptake in an in vivo adipocyte model (Kilgour et al. 1995) and this was due to reduced expression of both GLUT1 and GLUT4 in the plasma membrane (Smith et al. 1997; Takano et al. 2001). Muscle glucose uptake also decreases (Jessen et al. 2005) and could be due to the direct effect of GH on insulin action, or an indirect consequence of increased free fatty acid (FFA) flux (Møller and Jørgensen 2009). Increases in growth hormone, as well as epinephrine, also increase lipolysis, and consequently increased FFA concentrations (Schwarz et al. 2002; Kindermann et al. 1982; Rizza et al. 1982). The increase in circulating FFA interferes with glucose uptake via both insulin-dependent (Stefan et al. 2001; Segerlantz et al. 2003) and independent effects, which can act synergistically (Boden et al. 1994) by decreasing cellular glucose utilization (Ferrannini et al. 1983; Randle et al. 1963, 1964).

Given the complex interactions of systemic glucoregulatory factors governing glucose R_a_ and R_d_ in response to exercise, it may come as no surprise that there are discrepancies in studies assessing postprandial glucose tolerance in response to acute exercise. Some studies found glucose tolerance to remain unchanged (Roberts et al. 2013; Venables et al. 2007), while others reported glucose tolerance to be either increased (Mikines et al. 1988; Oberlin et al. 2014) or decreased (King et al. 1995; Rose et al. 2001; O'Connor et al. 2006). Whilst longer-term improvement in glycaemic control with exercise training is firmly established (Umpierre et al. 2013; Burgomaster et al. 2007; Mikus et al. 2012), there is also variability in the magnitude of the response (Boulé et al. 2005).

## Mediator to simulate or enhance the effects of exercise on glucose regulation

While duration (total training duration; acute session duration) and intensity are important considerations, findings from longer-term training studies (6 months) suggest that total work or energy expenditure is likely more important than either intensity or duration alone (Houmard et al. 2004). Increases in exercise “dose” (i.e., combination of exercise volume and intensity), however, can be difficult to achieve in some individuals due to (i) reduced adherence rates (Wadden et al. 1998); and (ii) increased risk for injuries (Girard et al. 2017; Fernández Menéndez et al. 2018). Not surprisingly, efforts have been made to identify the potential stimulus that may bypass the defects in insulin signalling in skeletal muscle. Alternative strategies, which may potentiate the effects of exercise at a reduced dose, have, therefore, been explored. One such strategy is exercise training in hypoxia (i.e., decreased oxygen concentration at the tissue level). Specifically, when exercise is performed in hypoxia at the same absolute intensity as in normoxia, the relative intensity (in relation to $\dot{V}{\text{O}}_{{2{\text{peak}}}}$) of exercise is higher compared to exercise in normoxia; which subsequently contributes to increased muscle glycogen utilization/depletion (Wadley et al. 2006; Parolin et al. 2000) and glucose *R*_d_ (Cooper et al. 1986). This is of particular relevance since skeletal muscle glycogen concentration is a potent regulator of insulin sensitivity and therefore, systemic glucose homeostasis (Shearer and Graham 2004). Furthermore, hypoxia may also stimulate the activation of signalling molecules (De Groote and Deldicque 2021), increasing glucose uptake similar to high-intensity exercise. As such, hypoxic exercise may represent an alternate strategy to enhance glycaemic control (via enhanced glucose metabolism). Accordingly, the following sections attempt to synthesize the effects of systemic hypoxia, with and without exercise on glucose homeostasis.

## Stimulation of glucose uptake by hypoxia

Although insulin and muscle contraction are the primary means to facilitate GLUT-4 translocation and increase glucose uptake, additional physiological stimuli including hypoxia can also increase glucose uptake (Fig. 1). For example, Cartee et al. (1991) showed that 3-methylglucose uptake in rat *epitrochlearis* muscle incubated under anoxic conditions (95% N_2_–5% CO_2_) for 40 min were approximately sixfold higher relative to baseline glucose uptake in oxygenated muscle (Cartee et al. 1991). Similarly, *rectus abdominis* muscle obtained from lean, obese and individuals with T2DM resulted in a twofold increase in glucose uptake relative to baseline when incubated under anoxic conditions (95% N_2_–5% CO_2_) (Azevedo et al. 1995). Several mechanisms may mediate GLUT4 trafficking in response to hypoxia, including an increase in intracellular Ca^2+^ concentration and activation of the downstream Ca^2+^/calmodulin-dependent protein kinase (CaMK); increase in reactive oxygen species; increased phosphorylation of AMPK via an increased AMP:ATP ratio (Mackenzie and Watt 2016; Sylow et al. 2021). These findings indicate that hypoxia stimulates glucose uptake via similar mechanisms to contraction-simulated pathways. Additionally, hypoxia-inducible factors (HIFs) are activated upon cellular exposure to hypoxia (Semenza 2011, 2012). HIF1ɑ has been implicated in the regulation of AKT activity (in human HepG2 cells) (Dongiovanni et al. 2008) and AMPK-mediated AS160 phosphorylation (Sakagami et al. 2014). These findings showing that hypoxia (similar to exercise) may stimulate glucose transport via pathways distinct from insulin imply that hypoxia may be a relevant strategy to improve glucose tolerance in individuals with insulin resistance.

## Hypoxia exposure under resting conditions on glucose tolerance and insulin sensitivity

Although in vitro experiments have shown that hypoxia stimulates glucose transport via activation of GLUT-4 translocation in the myocytes, it remains unclear if similar mechanistic pathways regulating glucose uptake and GLUT-4 translocation will be activated in vivo by hypoxia in humans. In one of the first studies to examine the effects of acute hypoxia (for 4 h) on intramuscular insulin signalling following a high glycaemic meal in healthy humans, D'Hulst et al. (2015) showed that (1) GLUT-4 in the sarcolemmal membrane was 30% higher in hypoxia compared to normoxia; (2) Rac1 and PAK1 were activated in normoxia but not hypoxia; and (3) hypoxia resulted in lower glucose response. Together, this result suggested that hypoxia may reduce glucose response to a high glycaemic meal by increasing the abundance of GLUT-4 at the sarcolemmal through insulin-independent pathways (D'Hulst et al. 2015).

However, while in vivo and in vitro studies have shown that hypoxia may elicit an increase in muscle glucose uptake, studies examining the effects of hypoxia on systemic glucose regulation have been inconsistent (Braun et al. 2001; Oltmanns et al. 2004; Mackenzie et al. 2011; D'Hulst et al. 2015). For instance, relative to normoxia, reduced glucose tolerance was observed when healthy men were exposed to hypoxia (by decreasing arterial oxygen saturation to 75%) for 30 min. Similarly, reduced insulin sensitivity has been reported in healthy men (Larsen et al. 1997) and women (Braun et al. 2001) following 48 h and 16 h of passive hypobaric hypoxic exposure (at 4559 m and 4300 m), respectively. In contrast, improved glucose tolerance has been observed following 60 min of passive hypoxia (FiO_2_ ~ 0.14), which was associated with a decrease in insulin AUC relative to passive normoxia. While some methodological differences exist between studies, these conflicting findings highlight the complex nature of the processes regulating the systemic glucose *R*_a_ and *R*_d_.

In this instance, evidence indicates that acute hypoxic exposure increases the hormonal responses of several glucoregulatory hormones that may augment *R*_a_ and *R*_d_, especially in individuals who are unacclimatized to a hypoxic environment (Moncloa et al. 1968). Most prominent among these glucoregulatory hormones is epinephrine; several studies have reported significant increases in epinephrine during hypoxic exposure which coincide with the development of insulin resistance (Larsen et al. 1997; Braun et al. 2001). In contrast, changes in glucagon, growth hormone and cortisol do not seem to be influenced by hypoxia (Larsen et al. 1997). However, studies have also observed an increase in cortisol (Woods et al. 2012; Moncloa et al. 1968); although cortisol concentration (being a stress hormone) may also be influenced by physiological stress when individuals ascent to high altitudes. As such, the dissociation between studies showing increased muscle glucose uptake and decreased systemic glucose tolerance following hypoxia could in part, be explained by changes in glucoregulatory hormones.

## Insulin and glucose response following acute exercise bout in hypoxia

Exercising in hypoxia (compared to normoxia) reduces oxygen availability and therefore induces a proportional shift in metabolic pathway flux (Davison et al. 2018). To compensate for the incomplete oxidation of glucose and reduced ATP-generating efficiency, exercise in hypoxia increases reliance on glucose and glycogen utilization (Larsen et al. 1997; Péronnet et al. 2006; Parolin et al. 2000). Although acute exercise in hypoxia induces a shift towards glucose and glycogen utilization, the subsequent effects on glycaemic control remain unclear (De Groote et al. 2021; Mackenzie et al. 2011, 2012a). Mackenzie et al. (2011) reported that an acute cycling bout (at an absolute intensity of 90% lactate threshold determined in normoxia) for 60 min in normobaric hypoxia (FiO_2_ ~ 0.146) enhanced insulin sensitivity in T2DM individuals to a greater extent when compared to exercise in normoxia (Mackenzie et al. 2011). In a subsequent study, improved insulin sensitivity was observed immediately following exercise in normoxia and hypoxia, although the effects appeared to be sustained 24 h post-exercise only after continuous cycling in hypoxia (Mackenzie et al. 2012b). In contrast, De Groote et al. (2021) showed that 60 min of continuous cycling (at a relative intensity based on heart rate corresponding to 55% $\dot{V}{\text{O}}_{2\max }$) in normobaric hypoxia (FiO_2_ ~ 0.14) increased (worsened) glucose AUC relative to baseline during an OGTT in both prediabetic and healthy individuals (De Groote et al. 2021). Despite exercising at the same relative intensity, cortisol levels during exercise in hypoxia were higher than in normoxia. Key regulators of the glucose transport pathways (e.g. AMPK, TBC1D1) were not reduced immediately after exercise by hypoxia (De Groote et al. 2021). These results suggest that hypoxia does not impair muscle insulin sensitivity locally, and the acute decrease in systemic glucose tolerance after exercise in hypoxia may be attributed to changes in the glucoregulatory hormones.

Given the systemic effects of hypoxia, it is possible that the interplay of glucoregulatory hormones, including glucagon, catecholamines, GH and incretin hormones could be disrupted, thereby altering glucose homeostasis. The extent of these increases in glucoregulatory hormones, however, depends on the severity and duration of hypoxia as well as the intensity of exercise in hypoxia relative to normoxia (absolute vs. relative intensity). In particular, exercise performed in hypoxia at the same absolute intensity as in normoxia has been shown to exaggerate the increase in epinephrine (Cooper et al. 1986). In contrast, submaximal exercise in hypoxia does not seem to alter the responses of catecholamines, glucagon and insulin compared to exercise in normoxia when performed at a similar relative intensity (Bouissou et al. 1986; Bailey et al. 2015; Engfred et al. 1994). Altogether, the identification of a single candidate hormone (e.g. epinephrine) that is altered by the addition of hypoxia to exercise, has so far been unable to account for the differences in acute systemic glucose tolerance (Larsen et al. 1997; Braun et al. 2001). Rather, it is more likely that several glucoregulatory hormones interact to induce the changes in systemic glucose tolerance (Eigler et al. 1979).

Exercise in hypoxia amplifies many intracellular processes associated with glucose uptake [e.g. GLUT-4 translocation, increased utilization of glycogen and glucose-derived metabolites Cooper et al. 1986; Cartee et al. 1991)]. As such, the glucose *R*_d_, at least into the contracting musculature, is expected to be increased. However, the localized increase in glucose uptake may not be reflected in the systemic glucose concentrations since a concomitant increase in the glucose *R*_a_ via hepatic glucose production and release are expected. Indeed, a nearly two-fold increase in the glucose *R*_a_ has been observed in hypoxia versus normoxia (Cooper et al. 1986). In addition to the effects of glucoregulatory hormones, the glucose *R*_a_ may also be influenced by circulating metabolites due to a shift in metabolic flux. Specifically, under hypoxic conditions, plasma triglyceride levels remain unchanged (De Groote et al. 2021) but increases when exercise is performed at the same relative intensity in normoxia. Additionally, the conversion of pyruvate to lactate is increased (Lundby and Van Hall 2002; Wadley et al. 2006), indicating an increase in glycolytic flux. Lactate, being a gluconeogenic substrate, constitutes a prime carbon source for the repletion of blood glucose and consequently muscle glycogen via the Cori cycle, while a proportion of accumulated lactate is also oxidized (Fournier et al. 2002, 2004). Further research is required to examine the contribution of lactate to glucose *R*_a_ following exercise in hypoxia. While the regulation of these metabolic pathways may reflect a transient acute response in maintaining metabolic balance to a perturbation (induced by hypoxia), longer-term training studies in hypoxia are needed to provide a greater understanding of any underlying metabolic changes.

## Effects of exercise training in hypoxia on insulin sensitivity

Findings from studies assessing the effects of exercise training in hypoxia on insulin sensitivity have been inconsistent (Haufe et al. 2008; De Groote et al. 2018; Wiesner et al. 2010; Klug et al. 2018). Haufe et al. (2008) showed that four weeks of training (60 min running, 3 days/week, at 3 mmol/L lactate value) in normobaric hypoxia (FiO_2_ 0.15) resulted in improvements in insulin sensitivity (assessed via HOMA index) and insulin AUC in healthy men. Additionally, De Groote et al. (2018) showed that six weeks of training (aerobic and resistance exercise, 3 days/week) in normobaric hypoxia (FiO_2_ 0.15), but not normoxia, reduced insulin and glucose AUC during an OGTT in obese adolescents. Importantly, whilst the absolute aerobic workload (power output during cycling multiplied by the duration of exercise) was lower in hypoxia, both normoxia and hypoxia training-induced similar improvements in insulin sensitivity (assessed via HOMA index). In contrast, six weeks of running (60 min running, 3 days/week, at 60% of $\dot{V}{\text{O}}_{2\max }$) did not alter glucose and insulin AUC in normobaric hypoxia (FiO_2_ 0.15) and normoxia in men with metabolic syndrome (Klug et al. 2018). Similarly, eight weeks of training (45 min cycling, 3 days/week, at 75% of maximal HR) in normobaric hypoxia (individually adjusted to SpO_2_ of 80%) and normoxia did not alter insulin sensitivity (assessed via HOMA index) in sedentary, overweight or obese individuals (Chacaroun et al. 2020); although $V{\text{O}}_{2\max }$ was significantly increased only following hypoxic training (~ + 10% vs ~  + 1%). However, it should be highlighted that individuals naïve to exercise training may elicit exaggerated responses to novel stimuli (hypoxia and/or exercise). Whether the larger improvements in insulin sensitivity following short-term (i.e., ≤ 6 weeks) hypoxia training in some studies was due to faster upregulation of selected metabolic markers, or whether the difference would have remained with longer training durations, remains to be determined.

Of note, studies reporting a comparable effect of exercise training on glucose tolerance in hypoxia and normoxia have employed similar relative exercise intensities (De Groote et al. 2018; Haufe et al. 2008). Accordingly, training in hypoxia is performed at a lower absolute intensity than in normoxia, which could be beneficial for individuals who are unable to tolerate high loads imposed on their locomotor system, including comorbidities such as knee osteoarthritis (Girard et al. 2017). However, given that the intensity (absolute *vs.* relative) of exercise largely influences skeletal muscle AMPK signalling (Wadley et al. 2006), muscle glycogen use (Wadley et al. 2006), as well as hormonal responses (catecholamines (Kjaer et al. 1988)), further investigation is required to determine the optimal method of implementing exercise intensity in hypoxia on insulin sensitivity.

A key concern associated with hypoxia training is the associated inflammatory response (Hosogai et al. 2007), given the widely established link between pro-inflammatory markers and insulin resistance (Petersen and Shulman 2018). An alteration in oxygen tension in the cells results in the activation of HIF, a key regulator of inflammation and immunity; whether the activation of HIF is pro- or anti-inflammatory in vivo is, however, dependent on the internal environment (Scholz and Taylor 2013). Additionally, it has also been suggested that hypoxia may trigger endoplasmic reticulum stress (Hosogai et al. 2007). That said, acute inflammatory responses are an important physiologic response that functions to restore tissue homeostasis and are required for beneficial adaptations (Medzhitov 2008). Accordingly, it is chronic or excessive inflammatory responses, that may impair metabolic regulation and that is associated with insulin resistance (Petersen and Shulman 2018). Further research will need to determine the role of hypoxic training in regulating the pro- and anti-inflammatory responses and the subsequent management of glycaemia in individuals with insulin resistance or T2DM.

## Hypoxic training—an integrated physiology approach

The hypothesis that hypoxic training may potentiate the effect of exercise on glucose tolerance is based on the findings that hypoxia activates glucose transport via pathways similar to muscle contraction (Kang et al. 1996), as well as the characterization of HIF1ɑ linking transcriptional responses to metabolic adaptation (Kierans and Taylor 2021). However, such a framework does not cover the conflicting effects of exercise in hypoxia on glycaemic control (Mackenzie et al. 2011; De Groote et al. 2021). As such, the role of the multiple, integrated systemic responses which regulate glucose tolerance need to be considered. Specifically, the balance between the net tissue glucose uptake and endogenous glucose production and release, are systemically regulated via the central nervous system (Güemes and Georgiou 2018), the endocrine system (Röder et al. 2016) and various inflammatory markers (Bruce and Dyck 2004); these factors sit on the backdrop of the individual (e.g. level of insulin sensitivity), the exercise variables (type, duration, intensity and frequency) as well as the hypoxic exposure (duration, severity of hypoxia, method of implementation [normobaric *vs.* hypobaric; intermittent *vs.* continuous]).

A clinical phenotype of insulin resistance and T2DM is a metabolic dysregulation of lipids, amino acids, and glucose. As such, analysis of the quantitative complement of metabolites in a biological system (i.e., metabolome) (Dunn et al. 2011) is a useful method to further the understanding of insulin resistance and T2DM. Indeed, metabolomics (i.e., the comprehensive study of all relevant metabolites in a biological system) has been extensively used in the study of T2DM (Milburn and Lawton 2013; Suhre 2014).

Molecular phenotyping has emerged as an essential tool in exploring, characterizing, and understanding the dynamic interactions between our genes and environment (diet, lifestyle) and their phenotypic expression across diverse human populations. While a wide array of biofluids, tissues and cells can be used in metabolic phenotyping studies, the collection of a blood sample is most frequently performed since it is minimally invasive. Analytical platforms for deep phenotyping of biofluids such as plasma, urine and cerebrospinal fluid based on nuclear magnetic resonance (NMR) spectroscopy and mass spectrometry (MS) can generate metabolic “fingerprints” that contain latent information relating to physiological or pathological status, informing on disease mechanisms, diagnosis and assessment of biological function (Belhaj et al. 2021; Dunn et al. 2011). As such, accurate measurement of hundreds of metabolites from a small amount of blood or urine sample (typically < 50 uL) can facilitate the identification of metabolites associated with T2DM, with metabolic profiling research already looking into branched-chain amino acids (Würtz et al. 2012; Tai et al. 2010), diacylglycerol (Wittenbecher et al. 2022), acylcarnitines (Sun et al. 2016) and ceramides (Haus et al. 2009). Accordingly, this has led to the discovery of biomarkers that may predict the onset and severity of T2DM. More recently, there has been an increased interest in the use of metabolic profiling approaches to identify molecular transducers mediating the metabolic benefits of exercise (Li et al. 2022). Importantly, the identification of mechanistic biomarkers could be used to determine the efficacy of hypoxic training stimulus. For instance, intermediate biomarkers could be used to predict the effectiveness of shorter-term training studies (6 weeks) instead of commonly used clinical endpoints (that determine the effectiveness of hypoxia training) such as $\dot{V}{\text{O}}_{2\max }$, HbA1c and glucose tolerance which may require a longer time frame to observe significant changes.

Another possible application of metabolomics is the analysis of metabolites associated with glycogen metabolism. As highlighted in this review, the depletion of glycogen following high-intensity exercise is an important factor promoting glucose transport and improving insulin sensitivity (Shearer and Graham 2004; Jensen et al. 2011). Within this context, the discovery of multiple proteins containing glycogen binding domains (regions within proteins that allow interactions with glycogen) has added increased complexity to the structure and processes regulating glycogen depletion and synthesis (McBride and Hardie 2009; Shearer and Graham 2004; Philp et al. 2012). The application of metabolomics and proteomics will likely progress our understanding of the processes regulating glycogen depletion and synthesis, including the apparent preservation of a minimum glycogen level in the skeletal muscle, and the link with insulin sensitivity.

A key challenge in hypoxic training is the inter-individual variation in response to hypoxia (Soo et al. 2020; Lawler et al. 2019). There is also heterogeneity in the metabolic profile (typically measured under fasting condition) of individuals with prediabetes (Chen et al. 2019). This is further complicated by the amplification of inter-individual variations in the metabolic profile when healthy humans are subjected to physiological stress (e.g. exercise, cold) (Krug et al. 2012). Accordingly, it is tenable to expect inter-individual variations in physiological responses when individuals with prediabetes undergo hypoxic training. In this instance, the use of metabolic profiling (using biomarkers that are known to be associated with T2DM) may help to discriminate individuals with prediabetes who are at higher risk of developing T2DM, that may require a more intensified training intervention (Stefan et al. 2015; Fritsche et al. 2021; Chen et al. 2019). Additionally, the hypoxia-induced metabolomic response can be measured to evaluate changes in metabolic profile due to the hypoxic training which may provide an early indicator of possible therapeutic benefits or harm of hypoxia.

## Conclusion

Systemic glucose regulation is intricately linked to cellular glucose transport, which is mediated by the translocation of glucose transport (i.e., GLUT-4) in response to a stimulus. The increase in glucose transport is not only influenced by insulin but can also be stimulated by muscle contraction. Importantly, glucose transport appears to be influenced by the exercise intensity. Unsurprisingly, high-intensity exercise is now widely accepted and recommended for individuals with T2DM (Mendes et al. 2016). While glucose uptake increases in the exercised skeletal muscle tissue, there remains a discrepancy between studies regarding the effects of acute exercise on systemic postprandial glucose tolerance. This may in part, be explained by different responses in glucoregulatory hormones (e.g. epinephrine, growth hormone, cortisol), which are in turn influenced by the intensity and duration of exercise. The increased rates of glucose uptake (disappearance) into the skeletal muscle with exercise, may thus have been negated by an increased rate of glucose appearance resulting in either unchanged or worsened systemic glucose concentration. Accordingly, the role of the multiple, integrated systemic responses which regulate glucose tolerance needs careful consideration to progress current knowledge.

The finding that contraction-induced increase in glucose uptake is not limited to exercise but can also be stimulated by hypoxia (Cartee et al. 1991) suggest that the exercise stimulus could be potentiated by hypoxia. The relative intensity of exercise (in reference to $\dot{V}{\text{O}}_{{{\text{2peak}}}}$) is substantially higher in hypoxia compared to normoxia at the same absolute workload. In this instance, current findings seem to indicate that exercise in hypoxia performed at the same relative intensity (i.e., lower absolute intensity) as in normoxia induces comparable effects on glucose tolerance. Hypoxic training may thus be an exercise intervention for individuals who are unable to tolerate high loads. However, further research is required to determine the optimal intensity during hypoxic training. Inter-individual variability in response to hypoxia may influence the outcomes of hypoxia training. The application of metabolomics presents as a promising approach to optimize hypoxic training by enabling the quantification of the individual metabolic responses to the training intervention and hypoxic stimuli.

## Data Availability

The data generated in the current study are available from the corresponding author on reasonable request.

## Abbreviations

AMP: Adenosine monophosphate
AMPK: AMP-activated protein kinase
ATP: Adenosine triphosphate
AUC: Area under curve
CaMK: Ca_2+_/calmodulin-dependent protein kinase
FFA: Free fatty acid
FiO_2_: Fraction of inspired oxygen
GAP: GTPase-activating protein
GH: Growth hormone
GLUT: Glucose transporters
HbA1c: Glycated haemoglobin
HIFs: Hypoxia-inducible factors
HIIE: High-intensity interval exercise
HIIT: High-intensity interval training
HOMA: Homeostasis model assessment
HOMA-IR: Homeostasis model assessment of insulin resistance
HR: Heart rate
kDa: Kilodalton
PI3K: Phosphatidylinositol 3-kinase
R_a_: Rate of glucose appearance
R_d_: Rate of glucose disappearance
SpO_2_: Arterial oxygen saturation
T2DM: Type 2 diabetes mellitus
$\dot{V}{\text{O}}_{2\max }$: Maximal oxygen uptake
$\dot{V}{\text{O}}_{{{\text{2peak}}}}$: Peak oxygen uptake
